# Hydroxycobalamin Reveals the Involvement of Hydrogen Sulfide in the Hypoxic Responses of Rat Carotid Body Chemoreceptor Cells

**DOI:** 10.3390/antiox8030062

**Published:** 2019-03-13

**Authors:** Teresa Gallego-Martin, Jesus Prieto-Lloret, Philip I. Aaronson, Asuncion Rocher, Ana Obeso

**Affiliations:** 1 Departamento de Bioquímica y Biología Molecular y Fisiología. Facultad de Medicina. Universidad de Valladolid. Instituto de Biología y Genética Molecular-CSIC, 47005 Valladolid, Spain; jesus.prieto@uva.es (J.P.-L.); rocher@ibgm.uva.es (A.R.); aobeso@ibgm.uva.es (A.O.); 2 Centro de Investigación Biomédica en Red de Enfermedades Respiratorias (CIBERES-ISCIII), 28029 Madrid, Spain; 3 Division of Pulmonary, Allergy and Critical Care Medicine, School of Medicine, University of Pittsburgh, Pittsburgh, PA 15213, USA; 4 Department of Inflammation Biology, School of Immunology and Microbial Sciences, Faculty of Life Sciences and Medicine, King’s College, London SE1 1UL, UK; philip.aaronson@kcl.ac.uk

**Keywords:** carotid body, hydrogen sulfide, hypoxia, hydroxycobalamin, oxygen sensing

## Abstract

Carotid body (CB) chemoreceptor cells sense arterial blood PO_2_, generating a neurosecretory response proportional to the intensity of hypoxia. Hydrogen sulfide (H_2_S) is a physiological gaseous messenger that is proposed to act as an oxygen sensor in CBs, although this concept remains controversial. In the present study we have used the H_2_S scavenger and vitamin B_12_ analog hydroxycobalamin (Cbl) as a new tool to investigate the involvement of endogenous H_2_S in CB oxygen sensing. We observed that the slow-release sulfide donor GYY4137 elicited catecholamine release from isolated whole carotid bodies, and that Cbl prevented this response. Cbl also abolished the rise in [Ca^2+^]_i_ evoked by 50 µM NaHS in enzymatically dispersed CB glomus cells. Moreover, Cbl markedly inhibited the catecholamine release and [Ca^2+^]_i_ rise caused by hypoxia in isolated CBs and dispersed glomus cells, respectively, whereas it did not alter these responses when they were evoked by high [K^+^]_e_. The L-type Ca^2+^ channel blocker nifedipine slightly inhibited the rise in CB chemoreceptor cells [Ca^2+^]_i_ elicited by sulfide, whilst causing a somewhat larger attenuation of the hypoxia-induced Ca^2+^ signal. We conclude that Cbl is a useful and specific tool for studying the function of H_2_S in cells. Based on its effects on the CB chemoreceptor cells we propose that endogenous H_2_S is an amplifier of the hypoxic transduction cascade which acts mainly by stimulating non-L-type Ca^2+^ channels.

## 1. Introduction

The carotid bodies (CB), located in the vicinity of the carotid artery bifurcations, are formed by clusters of two types of parenchymatous cells, chemoreceptor and sustentacular. Cell clusters are surrounded by a dense net of capillaries and penetrated by sensory nerve endings of the carotid sinus nerve (CSN) which form synaptic contacts with chemoreceptor cells; the soma of CSN fibres are located in the petrosal ganglion and project centrally to the nucleus tractus solitarius. Chemoreceptor cells sense the PO_2_, PCO_2_, and pH from nearby capillaries, becoming activated when PO_2_ decreases and/or PCO_2_/[H^+^] increases. Activation of chemoreceptor cells by either hypoxia or acidosis results in an increase of the ongoing basal normoxic/normohydric release of neurotransmitters leading to an increase in the action potential frequency in the CSN. Activity of the CSN is integrated in the brainstem to generate cardiorespiratory responses which act to restore blood gases to normal levels [1,2].

The chemoreceptor cell mechanisms linking the presentation of the stimulus to the activation of the exocytotic release of neurotransmitters, i.e., the sensory transduction cascades, are not fully understood. A commonly accepted transduction cascade for hypoxia consists of the following steps: detection of hypoxia by an oxygen sensor → coupling of the sensor to oxygen-sensitive K^+^ channels → change in K^+^ channel kinetics resulting in a decrease in their opening probability → chemoreceptor cell depolarization → activation of voltage-operated Ca^2+^ channels → increase in intracellular Ca^2+^ levels → exocytosis of neurotransmitters [2]. Several laboratories have recently proposed that hydrogen sulfide (H_2_S; hereafter referred to as sulfide as it exists as a mixture of H_2_S and HS^−^ at physiological pH) is an oxygen sensor, or an important positive regulator of the oxygen transduction cascade, in chemoreceptor cells [3,4,5,6,7,8,9,10]. Although Prabhakar’s laboratory argues that H_2_S is generated via activation of cystathionine-γ-lyase (CSE), according to a study by Li et al. [5] sulfide is produced by cystathionine β-synthetase (CBS). In contrast, Fitzgerald et al. [11] proposed that the increased rate of endogenous sulfide production that occurs during hypoxia would act as a brake to prevent chemoreceptor cells from becoming over-excited by intense hypoxic stimulus, and other laboratories have questioned the existence of any physiological role for endogenous sulfide in chemoreception, contending for example that the concentrations of sulfide donors (and therefore of sulfide) required to evoke either cellular effects at the CB level or reflex ventilatory effects are so high that their effects would represent an effect of cytochrome oxidase poisoning much like that of cyanide [12,13,14,15,16]. Thus, the physiological significance of endogenously produced sulfide in the transduction cascade of hypoxic stimulus in chemoreceptor cells is far from clear.

In order to further characterize the potential physiological significance of endogenously generated sulfide, in the present study we describe for the first time the use of the hydroxyl form of vitamin B_12_ or hydroxycobalamin as a new tool for studying the role of sulfide as a mediator of cell responses (hydroxycobalamin is protonated to H_2_O-cobalamin in physiological buffer, and we therefore refer to it as Cbl). The upper coordination bond of the cobalt in Cbl is made with a hydroxyl group which is displaced by H_2_S to form sulfhydrylcobalamin; the reaction is analogous to that occurring with HCN in forming cyanocobalamin. Cbl is therefore an effective and rapid sulfide scavenger; Van de Louw and Haouzi [17] showed that within 10 min of mixing 100 µM with an equimolar concentration of NaHS, the sulfide concentration in the solution fell by ~95%. These reactions constitute the foundation for the treatment of HCN poisoning; mice administered with i.p. Cbl were more than four times more likely to survive injection with an LD_85_ dose injection of NaHS [18], and Cbl is also used in humans for this purpose [19]. In vivo, vitamin B_12_ in the plasma is transported into cells when bound to the protein transcobalamin (TC II); the complex binds to a receptor (TCbIR/CD320) which is endocytosed [20] and then degraded in lysosomes, releasing the Cbl into the cytoplasm where it is converted into methylcobalamin. Cbl is also transported into the mitochondria, where it is converted to 5′-deoxyadenosyl-cobalamin [21]. Less is known about the uptake of Cbl in the absence of TC II, although Berliner and Rosenberg demonstrated that platelets were able to take up appreciable amounts of vitamin B_12_ in the absence of transcobalamin via a separate process [22]. Hall et al. (1979) also showed that HeLa cells were able to take up free vitamin B_12_ in the absence of transcobalamin and that this led to the synthesis of both methyl- and 5′-deoxyadenosyl-cobalamin, suggesting that the vitamin B_12_ had entered both the cytoplasm and the mitochondria [23]. More recently, studies carried out in thymocytes [24] and synaptosomes [25] provide evidence that cyanocobalamin, which has a similar structure to hydroxycobalamin, is able to cross the plasmalemma in the absence of TC II when applied in vitro at supraphysiological concentrations.

Therefore, it would be predicted that Cbl, if applied before H_2_S donors, should suppress their effects. More importantly, Cbl should scavenge sulfide produced by CSE, CBS, and other enzymes, which are present in the cytoplasm and/or the mitochondria [26]. Employing a sulfide scavenger has several potential advantages over using current methods designed to reduce cellular [sulfide] in order to evaluate its effects, particularly with regard to hypoxia. For example, it is possible that blocking or knocking out the enzymes that synthesize sulfide does not prevent an increase in its cellular concentration during hypoxia. Since there are multiple sources of sulfide within cells, both enzymatic and non-enzymatic [26], and since hypoxia is proposed to increase the cellular [sulfide] by blocking its metabolism [10], its concentration might still rise under reduced oxygen conditions if only one of these sources is removed. Additionally, the use of mice in which CSE, thought to be primarily responsible for sulfide production in the cardiovascular system, has been knocked out has been criticized because these animals demonstrate a striking rise in plasma homocysteine [27]. In contrast, Cbl would be predicted to prevent a rise in sulfide, regardless of its cause, without causing toxicity.

Accordingly, we examined whether two H_2_S donors, the classical sodium hydrogen sulfide (NaHS) and the more physiological releaser GYY4137, activated catecholamine release by freshly isolated rat CBs in a fully Cbl-sensitive manner, as well as whether Cbl blocked catecholamine release caused by different levels of hypoxia and high K^+^-evoked depolarization. The results demonstrated that Cbl ablated responses to both GYY4137 and NaHS and inhibited hypoxia-induced catecholamine release elicited at several levels of hypoxia ~80% in freshly isolated intact CBs but was ineffective in modifying the release response elicited by 25, 35, and 60 mM extracellular K^+^. In isolated chemoreceptor cells, Cbl also abolished the rise in [Ca^2+^]_i_ elicited by NaHS, inhibited the hypoxia-induced Ca^2+^ response ~70%, and left intact the response elicited by high K^+^. We conclude that endogenous H_2_S has a specific physiological action in enhancing hypoxic responses in chemoreceptor cells; this may involve mainly the stimulation of T-type voltage-gated Ca^2+^ channels. Some preliminary data from this study were presented at the XIXth meeting of the International Society for Arterial Chemoreception held in Leeds in July 2014 [28].

## 2. Materials and Methods

### 2.1. Animals and Anesthesia Surgical Procedures

Experiments were performed using CBs from adult male Wistar rats (280–350 g body weight). Animals were anaesthetized with sodium pentobarbital (60 mg/kg, i.p.) dissolved in physiological saline, and then euthanized by an intracardiac overdose of sodium-pentobarbital. In handling the animals, we followed the European Union Directive for Protection of Vertebrates Used for Experimental and Other Scientific Ends (2010/63/EU). Protocols were reviewed and approved by the University of Valladolid Institutional Committee for Animal Care and Use (Project Approval Ethical Code: 4505502)

After tracheostomy, bilateral blocks of tissue containing the carotid bifurcations were removed and placed in a dissecting chamber filled with ice-cold O_2_-saturated Tyrode solution (in mM: NaCl, 140; KCl, 5; CaCl_2_, 2; MgCl_2_, 1.1; HEPES, 10; glucose, 5.5; pH = 7.4). The CBs were cleaned of surrounding tissue under a dissecting microscope and saved in glass vials containing O_2_-saturated ice-cold Tyrode for use within the next hour.

### 2.2. Isolation and Chemoreceptor Cell Culture

CBs were incubated (12 min; 37 °C) in nominally Ca^2+^- and Mg^2+^-free Tyrode’s solution (pH = 7.2) containing collagenase (2.5 mg/mL, type IV, Sigma) and bovine serum albumin (BSA; 6 mg/mL, Fraction V, Sigma). After removing the solution, the CBs were incubated for 17 min period in a new Tyrode solution containing trypsin (1 mg/mL, type II, Sigma) and BSA (6 mg/mL). The trypsin-containing solution was removed, and the tissues were mechanically disrupted by aspiration through a P1000 pipette in 2 mL of culture medium (DMEM) supplemented with 10% fetal bovine serum, 2 mM L-glutamine, and 1% penicillin/streptomycin/fungizone. After centrifugation (2000 rpm, 7 min), the supernatant was discarded, and the cell pellet was resuspended in 100 µL of fresh culture medium. Dispersed cells were plated as 10–20 µL drops on small poly-L-lysine-coated coverslips kept in 12 well plates and maintained in a humidified incubator (37 °C; 5% CO_2_ in air). Once the cells attached (45 min after plating), 1.5 mL of DMEM was added into each well to maintain the cells until use (16–24 h later).

### 2.3. H-Catecholamine (^3^H-CA) Release Experiments Using Intact CBs

General procedures used to label chemoreceptor cell CA deposits and to later study their release have been described in previous publications [29]. Analytical methods have previously been described in detail [30]. In brief, CBs were incubated (2 h; 37 °C) in Tyrode solution containing the CA precursor, ^3^H-tyrosine, of high specific activity (40–50 Ci/mmol; Perkin-Elmer), 6-methyl-tetrahydropterine (100 µM), and ascorbic acid (1 mM). Following ^3^H-CA labeling, individual CBs were transferred to glass vials provided with caps, and with an inlet for the gas line and an outlet to avoid a pressure build-up, which contained 2 mL of precursor-free Tyrode bicarbonate solution (composition as above except for the substitution of 24 mM NaCl by 24 mM NaHCO_3_). Initial incubation in precursor-free normoxic (5% CO_2_/20% O_2_/75% N_2_) solution lasted 1 h, with solutions renewed every 20 min and discarded; these incubations were made to wash out the precursor and to eliminate the readily releasable ^3^H-CA pool [30]. Thereafter, the incubating solutions (2 mL) were renewed every 10 min and collected for the analysis of their ^3^H-CA content. Specific protocols are given in the Results section. Solutions of NaHS and Cbl hydrochloride (both from Sigma-Aldrich) and (*p*-methoxyphenyl)-morpholino-phosphinodithioic acid (GYY4137; Cayman Chem. Co.) were freshly prepared as stock solutions and maintained in capped vials at 0–4 °C until use. ^3^H-CA concentrations of collected incubation solutions as well as CB homogenates were analyzed as described previously [30].

### 2.4. Intracellular Ca^2+^ Measurements

Detailed descriptions of the recording procedures and equipment can be found in previous publications [31,32]. Briefly, cells on coverslips were treated with 10 µM fura-2-acetoxymethyl ester (Molecular Probes) at room temperature (20–24 °C) for 30 min and washed for 30 min, and coverslips were then mounted in a perfusion chamber placed on the stage of a Nikon Diaphot 300 inverted microscope. Cells were superfused through gas impermeable lines with pre-warmed (37 °C) Tyrode bicarbonate equilibrated with 5% CO_2_/20% O_2_/75% N_2_ (except when hypoxia was used as a stimulus), which was reheated to 37 °C at the entrance of the recording chamber. High K^+^ solutions were prepared by replacing equimolar amounts of NaCl by KCl. To apply NaHS, perfusing solutions were equilibrated with 20% O_2_/5% CO_2_/balance N_2_) at 37 °C and the reservoirs were capped. Immediately before the perfusion, the sulfide donor was added from a freshly prepared stock that was maintained at 0–4 °C.

### 2.5. Statistics

All data are expressed as the mean ± S.E.M. Statistical analysis were performed using the two-tailed Student *t*-test for unpaired data and RM one-way ANOVA, with the Greenhouse–Geisser correction and Sidak’s multiple comparisons test for the Ca^2+^ imaging experiments. Values of *p* < 0.05 were considered to indicate statistical significance.

## 3. Results

### 3.1. Effects of Cbl on the Release of ^3^H-CA Induced by Hydrogen Sulfide Donors

**Figure 1:**
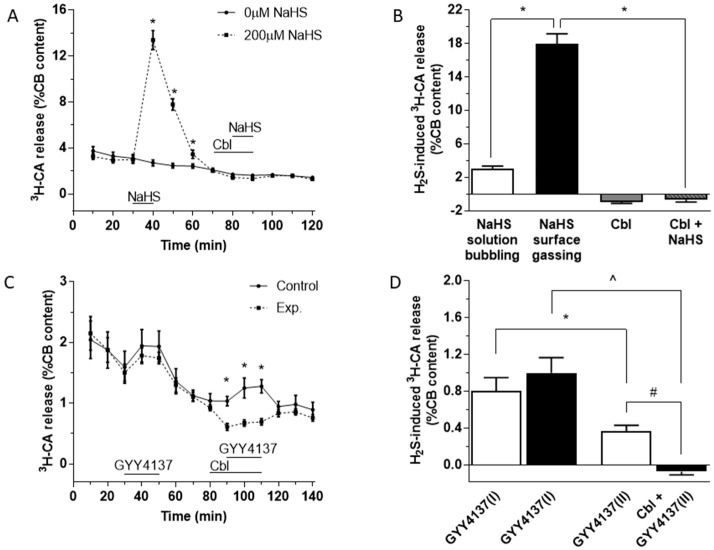
Effect of NaHS and GYY4137 on the release of ^3^H-CA by CB chemoreceptor cells in the presence and absence of Cbl. (**A**) Time course of catecholamine release elicited by 200 µM NaHS applied for 10 min. A second application of NaHS was in the presence of 300 µM Cbl (n = 6 and 8, for 0 and 200 µM NaHS, respectively). (**B**) The black bar shows the magnitude of the release response corresponding to the area under the curve in part A. The empty bar represents the release response obtained for 200 µM NaHS in similar experiments in which the solution rather than the headspace was gassed. The small columns in grey at the right of the figure show that 300 µM Cbl abolished the effect of NaHS, and that when applied alone Cbl caused a modest inhibition of the basal ongoing release of catecholamine (NaHS solution bubbling, n = 2; NaHS gassing surface, n = 8; Cbl, n = 18; Cbl + NaHS, n = 10). (**C**) Effect of GYY4137 on the release of ^3^H-CA in the absence and presence of Cbl. 400 µM GYY4137 was applied for 20 min by itself at 30 and 90 min (continuous line); 400 µM GYY4137 was applied for 20 min by itself at 30 min and then again at 90 min, this time in the presence of 300 µM Cbl, which had been applied at 80 min (dashed line). n = 10 in each condition. (**D**) The bars show the amplitudes of the two successive responses to GYY4137 (area under the curve) when both responses were evoked in the absence of Cbl (empty bars) and when the second response was elicited in the presence of Cbl. Symbols indicate where there was a significant effect.

As depicted in Figure 1A, application of 200 µM NaHS for 10 min (between 30 and 40 min on this protocol; dashed line) caused a large and reversible increase in ^3^H-CA release. After allowing the effect of sulfide to subside, 300 µM Cbl was then applied between 70 and 90 min (for 20 min). In the presence of Cbl, adding 200 µM NaHS to the solution (between 80 and 90 min) caused no increase in ^3^H-CA release. In the absence of sulfide, application of Cbl on its own caused a small fall in catecholamine release (solid line). Figure 1B summarizes the findings of these experiments; note that the substantial response elicited by NaHS was completely eliminated when Cbl was present, and that application of Cbl on its own caused a small fall in ^3^H-CA release.

In the experiments shown in Figure 1A the incubating solutions were pre-equilibrated at 37 °C with a water vapor-saturated gas mixture (20% O_2_/5% CO_2_/75% N_2_), and throughout the experiments the headspace in the vials above the solutions was continually gassed. We also examined the effect of 200 µM NaHS on ^3^H-CA release using an identical protocol to that shown in Figure 1A, except that the solution itself rather than the headspace above it was bubbled with the same gas mixture. As shown in Figure 1B (white bar) the effect of applying 200 µM NaHS was greatly diminished if the solution itself was bubbled. This observation would imply that bubbling the solutions directly causes the outgassing of H_2_S released into the solution by NaHS, and in fact a similar phenomenon occurs when NaCN is used as a stimulating agent [33]. Therefore, in the subsequent ^3^H-CA release experiments described below, the equilibrating gas mixture was always applied to the vials above the surface of the incubating solution in an effort to reduce the loss of sulfide from the solution.

An important criticism of experiments using NaHS as a sulfide donor is that the concentrations required to obtain cellular responses are typically high and, in addition, that the time course of H_2_S release from NaHS is nearly instantaneous. Conversely, the production of endogenous H_2_S by enzyme-catalyzed reactions in cells is likely to occur in lesser amounts, at a much slower rate, and more steadily. Therefore, using NaHS may not provide a good model for the biological effects of naturally produced H_2_S [34]. To address this issue, several new generations of sulfide donors have been synthesized [35]. Among them (*p*-methoxyphenyl)-morpholino-phosphinodithioic acid (GYY4137) has been proven to release sulfide at a much lower rate and with a long-lasting time course generating cell responses considered to be more physiologically relevant [36]. When tested in the CBs (Figure 1C), GYY4137 (400 µM) indeed caused a small but significant ^3^H-CA release. A response to GYY4137 was also observed during a second application (Figure 1C, solid line), although this was smaller than the first response (Figure 1C, black line; Figure 1D, II vs. I). This second response was abolished by Cbl (Figure 1C, dashed line). In addition, an inhibitory effect of Cbl on basal ^3^H-CA release is apparent at the 90 min point just prior to the application of GYY4137, implying that that endogenous sulfide production contributes to the genesis of basal normoxic release of ^3^H-CA. These results are quantitated in Figure 1D.

### 3.2. Effects of Cbl on the Release of ^3^H-CA Induced by Hypoxic and High External K^+^ Stimulation of the Carotid Body

**Figure 2:**
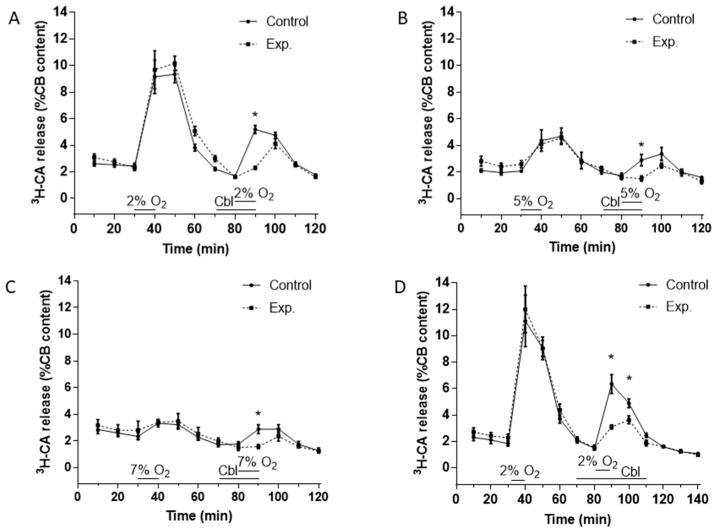
Effects of Cbl on ^3^H-CA release response elicited by different levels of hypoxia. The continuous lines in (**A**), (**B**), and (**C**) show the effects on catecholamine release of successive exposures of CBs (at 40–50 min, SI, and 80–90 min, SII) to 2% O_2_ (**A**, n = 12 in each group), 5% O_2_ (**B**, n = 5 in each group), and 7% O_2_ (**C**, n = 5 in each group) under control conditions, whereas the dashed lines illustrate catecholamine release evoked by a similar protocol when 300 µM Cbl was present between 70 and 90 min. (**D**). Protocol similar to panels A except that Cbl was present both during and after the second hypoxic challenge (from 70 to 110 min), n = 8 and 12, control and experimental group, respectively. Asterisks connote time points where there were significant differences between the amplitudes of ^3^H-CA release observed in CBs which were exposed or not exposed to Cbl.

Figure 2A–C show the effects of Cbl on the release of ^3^H-CA induced by hypoxic stimuli of three different intensities. Stimuli consisted of the incubation of the CBs for 10 min in solutions equilibrated with gas mixtures containing 2% O_2_ (PO_2_ ≈ 13 mmHg), 5% O_2_ (PO_2_ ≈ 33 mmHg), and 7% O_2_ (PO_2_ ≈ 46 mmHg). As shown in Figure 2A–C, for each intensity of hypoxia, both control and experimental CBs were stimulated twice with the hypoxic stimulus. The controls were stimulated twice (at 30–40 and 80–90 min) with hypoxia alone, whereas the experimental CBs were stimulated first with hypoxia alone at 30–40 min and then at 80–90 min following a 10 min preincubation with 300 μM Cbl, which was also present throughout the hypoxic challenge. This experimental design allowed us to calculate the ratio of the release response in the second (SII) to the first (SI) presentation of the stimulus for both control and experimental CBs (see below).

In every case (Figure 2A–C), it is evident that in drug-free hypoxic stimulations the release response elicited by hypoxia developed fully during the 10 min hypoxic period, was maintained at a similar level over the next 10 min, and then fell progressively over the subsequent two or three normoxic incubating periods that followed. When present during only the hypoxic stimulus, Cbl markedly depressed the component of the ^3^H-CA release response measurable during hypoxia, but seemed to have no significant effect on release during the normoxic incubating periods that followed. This pattern of the inhibition prompted a further group of experiments using the most intense hypoxic stimulus (2% O_2_) in which Cbl was applied 10 min prior to and throughout the 10 min of hypoxia, and then also during the two 10 min normoxic periods that followed. The results of these experiments are shown in Figure 2D.

**Figure 3:**
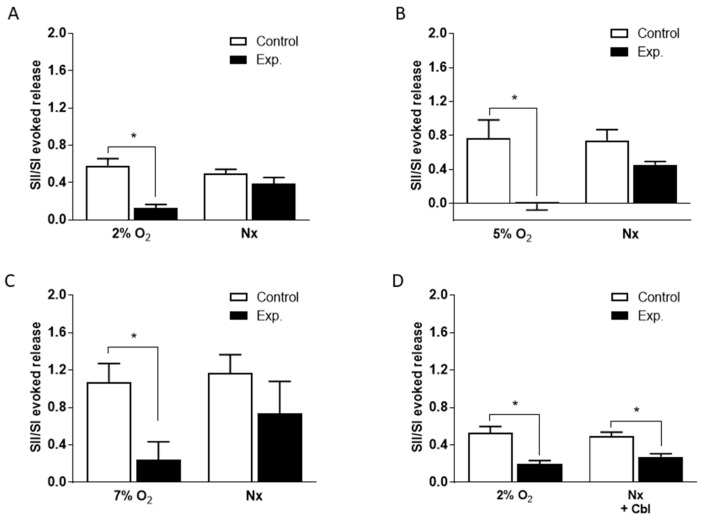
Mean effects of 300 µM Cbl on the ratio of the second and first ^3^H-CA responses to hypoxia shown in Figure 2. Results were calculated separately for ^3^H-CA release during hypoxia and during the subsequent 20 min of normoxia (Nx) during which release fell back to the baseline. (**A**)–(**C**) Mean effects of Cbl on the ratio of the second and first responses to hypoxia for 2, 5, and 7% O_2_; Cbl was present for 10 min before and during the second hypoxic period. (**D**) Mean effects of Cbl on the ratio of the second and first responses to hypoxia for 2% O_2_; Cbl was present for 10 min before and during the second hypoxic period, as well as during the subsequent 20 min of normoxia. Asterisks indicate where there was a significant effect of Cbl. Number of replicates for each condition as is indicated in Figure 2.

In order to quantitate the effects of Cbl shown in Figure 2, we calculated the SII/SI ratios in the absence and presence of the vitamin B analog, during the period of hypoxia and during the subsequent normoxic period during which ^3^H-CA release was falling back to the basal level. These results are presented in Figure 3. As shown in Figure 3A–C, when Cbl was present before and during hypoxia, but not afterwards, it very strongly suppressed the release of ^3^H-CA evoked by each level of hypoxia but had no significant effect on catecholamine release during the subsequent period of normoxia. On the other hand, as shown in Figure 3D, if Cbl was left in the solution for the 20 min following hypoxia, it suppressed ^3^H-CA release both during hypoxia, and afterwards. These results are consistent with the possibility that elevations in chemoreceptor cell sulfide levels may persist transiently following hypoxia, slowing the recovery of baseline catecholamine release.

**Figure 4:**
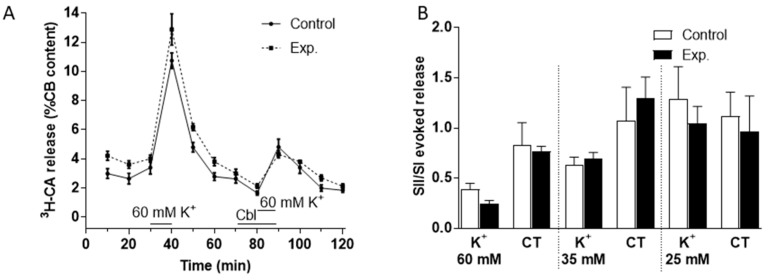
Effects of Cbl on the ^3^H-CA release response elicited by three elevated levels of extracellular K^+^. (**A**) The continuous line in shows the effects on catecholamine release of successive exposures of CBs (at 30–40 min, SI, and 80–90 min, SII) to 60 mM K^+^ (n = 5 in each condition) under control conditions, whereas the dashed lines illustrate catecholamine release evoked by a similar protocol when 300 µM OH- Cbl was present between 70 and 90 min. (**B**) Bars illustrate the ratio of the second and first responses to high K^+^ in the presence and absence of Cbl during the 10 min of high K^+^ and during the subsequent 20 min period in control Tyrode (CT) solution (K^+^ 5mM) during which catecholamine release remained elevated with 60, 35, and 25 mM (n = 5 in each condition).

We used a similar protocol to investigate the effects of Cbl on ^3^H-CA release evoked by high external K^+^ (60 mM, see Figure 4A; 35 and 25 mM, not shown) and found that the effect of these purely depolarizing stimuli was not obviously affected by 300 µM Cbl. Analysis of the effects of Cbl on catecholamine release both during the presence of K^+^ and subsequent recovery period in control Tyrode solution was carried out separately as described above for hypoxia. As illustrated in Figure 4B, this confirmed that there was no significant effect of Cbl during either period. Likewise, a further analysis in which ^3^H-CA release was summed over both periods revealed no significant effect of Cbl (not shown).

### 3.3. Effects of Cbl on the Intracellular Calcium Transients Induced by NaHS, Hypoxia, and High External K^+^

**Figure 5:**
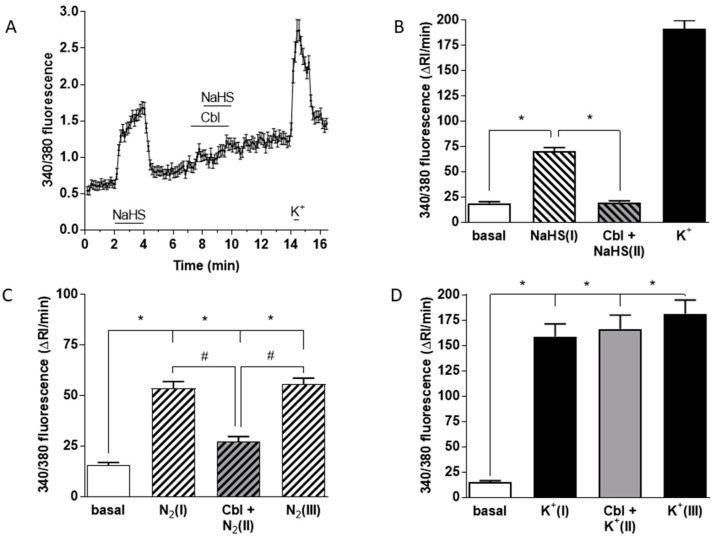
Effects of Cbl on the intracellular Ca^2+^ responses elicited by 50 µM NaHS, hypoxia, and high external K^+^. [Ca^2+^]_i_ was assessed as the F340/F380 fluorescence emission ratio in isolated chemoreceptor cells loaded with fura-2. (**A**) Mean +/− SEM 340/380 fluorescence ratio (calculated every 8 s) from 74 cells stimulated with NaHS in the absence and presence of 300 µM Cbl. The response to high K^+^ at the end demonstrates cell viability. The fast Ca^2+^ transient in response to high K^+^ at the end of experiments demonstrates that cell was viable. (**B**) Mean running integrals of the basal and stimulated fluorescence signals (Δ fluorescence/min) obtained from the 74 chemoreceptor cells recorded as in (A). (**C**) Mean running integrals of the fluorescence signals obtained in 43 cells recorded following the sequence depicted in the figure consisting of the application of three identical hypoxic stimuli (perfusion with 5% CO_2_/95% N_2_) except that the middle hypoxic challenge was applied in the presence of 300 µM Cbl. (**D**) Mean values obtained in 44 cells in a similar ‘sandwich’ style experiment using high (35 mM) external K^+^ as the stimulus. Symbols connote significant differences between the columns indicated.

Figure 5A shows mean +/− SEM 340/380 fluorescence ratio from 74 cells of the Ca^2+^ transients elicited in chemoreceptor cells by 50 μM NaHS under normoxic conditions. In contrast, the same concentration of the sulfide donor in the presence of 300 μM Cbl had no effect on [Ca^2+^]_i_. A subsequent pulse of high K^+^ (35 mM) to assess the responsiveness of the cells to depolarization induced the expected rise in [Ca^2+^]_i_.

The averaged Ca^2+^ signal obtained from 74 cells subjected to this protocol reveals that neither Cbl itself nor 50 mM NaHS applied in the presence of Cbl had a significant effect on [Ca^2+^]_i_. Figure 5B shows mean running integrals of the fluorescence signals (Δ fluorescence/min) [31] obtained in these chemoreceptor cells when exposed to these conditions. It is evident that Cbl abolished the Ca^2+^ response elicited by 50 μM NaHS. Figure 5C shows the mean results obtained in 43 chemoreceptor cells using hypoxia as a stimulus. Experiments were performed following a sandwich-type protocol [32]: after several minutes of recording under normoxic conditions, a first hypoxic stimulus (perfusing solutions pH 7.4 bubbled with 5% CO_2_–95% N_2_; peak hypoxia in the recording chamber ≈ 10 mmHg; [N_2_(I)] in Figure 5C) for 2 min was applied. Following a subsequent 5–8 min period of recording under normoxic conditions, we started perfusion with a normoxic solution containing 300 μM Cbl for 1 min, immediately followed by a second hypoxic stimulus ([Cbl + N_2_(II)]). After an additional normoxic recovery period in the absence of Cbl, a final hypoxic stimulus ([N_2_(III)]) was then applied. It is evident that Cbl reversibly inhibited the hypoxia-induced Ca^2+^ signal by ~70%. In an identical group of experiments using 35 mM K^+^ as a stimulus in 45 chemoreceptor cells (Figure 5D), application of an analogous protocol demonstrated that Cbl did not affect the rise in [Ca^2+^]_i_ induced by the pure depolarizing stimulus.

**Figure 6:**
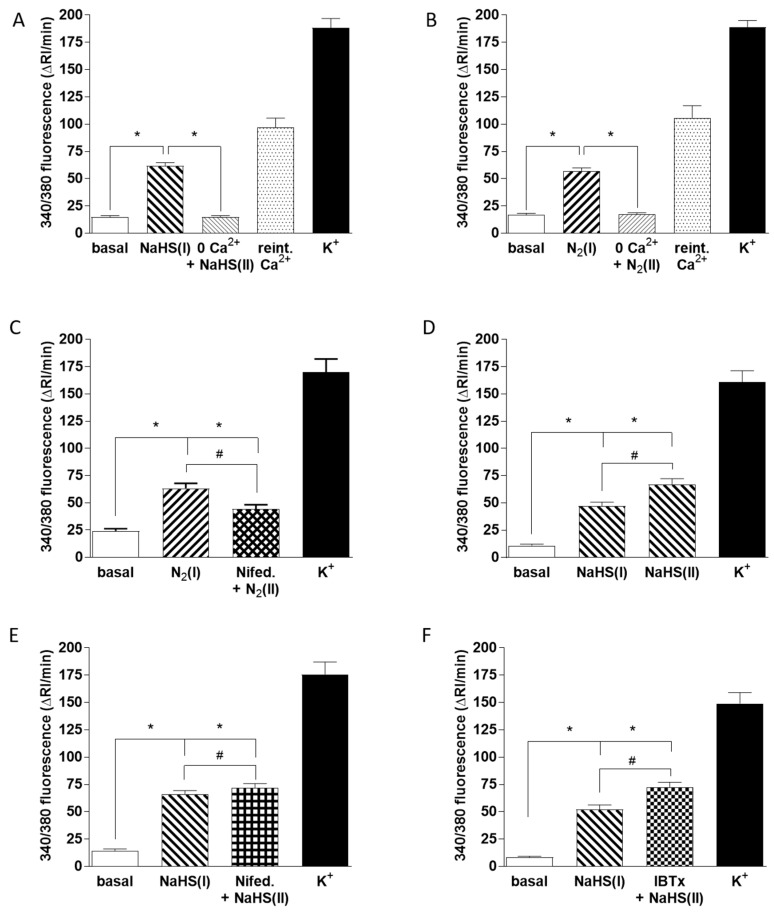
Mechanisms of Ca^2+^ influx induced by hypoxia and 50 µM NaHS. [Ca^2+^]_i_ was assessed as the F340/F380 fluorescence emission ratio in isolated chemoreceptor cells loaded with fura-2. In all cases the protocols followed the same pattern: a first exposure to hypoxia or NaHS, subsequent application of the experimental condition 0 mM Ca^2+^ (1 min, **A**,**B**), 2 µM nifedipine (3 min, **C** and **E**), or 100 nM IBTx (1 min, **F**) followed by that condition with hypoxia or NaHS and finally high K^+^ to demonstrate that cells were viable. Bars in all panels indicated the running integrals of the fluorescence signals (Δ fluorescence/min) obtained during the various conditions. (**A**) Effect of Ca^2+^ free solution on the Ca^2+^ signal caused by NaHS (n = 66 cells). (**B**) Effect of Ca^2+^ free solution on the Ca^2+^ signal evoked by hypoxia (n = 44 cells). In both cases, Ca^2+^ reintroduction caused a rebound increase in the fluorescence emission ratio. (**C**) Effect of nifedipine on the Ca^2+^ signal elicited by hypoxia (n = 24 cells). (**D**) Ca^2+^ signals evoked by two successive exposures of cells to NaHS under control conditions, data from 51 cells. (**E**) Ca^2+^ signals evoked by two successive exposures of cells to NaHS—the first under control conditions and the second in the presence of nifedipine (n = 50 cells). (**F**) Ca^2+^ signals evoked by two successive exposures of cells to NaHS; the first under control conditions and the second in the presence of IBTx (n = 47 cells). Symbols connote significant differences between the columns indicated.

In further experiments we compared the mechanisms by which hypoxia and NaHS were causing a rise in CB cell [Ca^2+^]_i_. As shown in Figure 6A,B removal of Ca^2+^ from the solution caused a similar and profound suppression of the Ca^2+^ transients elicited by both 50 µM NaHS and hypoxia (measured in 66 and 48 chemoreceptor cells, respectively). This was consistent with the well-established role of Ca^2+^ influx via voltage-gated channels in the hypoxic response [37] and indicated that the rise in [Ca^2+^]_i_ caused by NaHS was also due to Ca^2+^ influx. As shown in Figure 6C, a component of the Ca^2+^ influx response to hypoxia, measured in 24 cells, was blocked by the L-type voltage-gated Ca^2+^ channel blocker nifedipine (2 µM). Whereas two successive hypoxia-induced Ca^2+^ transients were similar in size under control conditions (Figure 5C), as shown in Figure 6D, the rise in [Ca^2+^]_i_ elicited by 50 µM NaHS tended to increase during the second exposure to this sulfide donor (51 chemoreceptor cells). This increase was significantly smaller when 2 µM nifedipine was present during the second response (Figure 6E; measured in 50 cells), indicating that this drug was also blocking the response to sulfide (28% inhibition). However, nifedipine diminished the response to hypoxia to a significantly greater extent than it did the response to NaHS (compare Figure 6D,E).

As it has been proposed that sulfide-induced inhibition of BK_Ca_ channel activity in CBs may be important in O_2_ sensing [38], we also examined the effect of the BK_Ca_ channel blocker iberiotoxin (IBTx; 100 nM) on the increase in chemoreceptor cell [Ca^2+^]_i_ caused by sulfide (47 chemoreceptor cells). As shown in Figure 6F, IBTx had no effect on this response (compare Figure 6D,F).

## 4. Discussion

The main findings of this study can be summarized as follows: (1) the hydrogen sulfide donors NaHS and GYY4137 stimulated ^3^H-CA release, and this response was abolished by 300 µM Cbl; (2) Cbl diminished the hypoxia-induced release of ^3^H-CA by 75–100%, and eliminated and reduced the intracellular Ca^2+^ transients elicited by 50 µM NaHS and hypoxia, respectively, by ~70%; (3) in contrast, neither the release of ^3^H-CA nor the Ca^2+^ transients elicited by high external K^+^ were affected by Cbl; (4) nifedipine caused a relatively small inhibition in the rise in CB chemoreceptor cells [Ca^2+^] elicited by sulfide whilst attenuating the Ca^2+^ signal caused by hypoxia to a greater extent; (5) IBTx had no effect on the Ca^2+^ transients elicited by NaHS in chemoreceptor cells. The physiological forms of vitamin B_12_ in mammalian cells are methylcobalamin and 5′-deoxyadenosyl-cobalamin, with hydroxycobalamin being one of the most abundant forms of vitamin B_12_ in the diet. Cyanocobalamin and hydroxycobalamin are the forms of vitamin B_12_ used most commonly for therapy. The cellular uptake of vitamin B_12_ from the blood depends on its binding to the plasma protein transcobalamin II (TC II); this complex binds to a membrane receptor (TCbIR/CD320), which is then endocytosed [20]. Released when the endocytosed receptor is processed in lyososomes, vitamin B_12_ is methylated in the cytoplasm and, also in its hydroxycobalamin form, is transferred to mitochondria to form 5′-deoxyadenosyl-cobalamin [21]. This uptake process is Ca^2+^- and energy-dependent, and is saturable with an affinity constant in the subnanomolar range [20], which is appropriate in that the total plasma concentration of vitamin B_12_ is ~0.3 nM, with about 25% of this bound to TC II [39]. However, there is evidence that vitamin B_12_ uptake into cells can occur via other pathways in vitro, and even in vivo, especially at high concentrations. For example, complete congenital TC II deficiency can be treated with very high doses of cobalamin [40]. Of more direct relevance to our work, a study of the uptake of cyanocobalamin in cultured platelets, carried out in serum-free physiological saline, demonstrated the existence of a non-saturating and Ca^2+^-independent component of vitamin B_12_ uptake, which occurred even in TC II-deficient platelets [22]. The ability of Cbl to enter cells in vitro was also suggested in a study [18] in which freshly isolated hepatocytes were incubated for 30 min in physiological saline solution containing NaHS in sealed vials, and 100 µM Cbl was applied either at the same time as sulfide or 20 min subsequently. In both cases, the cytotoxic effects of sulfide were almost abolished. The observation that Cbl was able to exert its full protective action when added well after NaHS suggests strongly that it was able to enter cells and scavenge sulfide within them, as well as remove it from the incubation medium. Additional in vitro studies [24,25] using cyanocobalamin at supraphysiological levels (10–1000 µM) comparable to the concentration we used in our experiments (300 µM) also suggest strongly that this form of vitamin B_12_ is able to cross the plasmalemma and impact cell function in the absence of TC II. In both cases, cyanocobalamin raised the intracellular Ca^2+^ concentration; in thymocytes this was shown to be due mainly to inhibition of Ca^2+^ uptake by the endoplasmic reticulum [24], whilst in synaptosomes it appeared that the rise in [Ca^2+^]_i_ was mostly dependent on activation of N/P/Q type Ca^2+^ channels via a PKC-dependent mechanism [25]. In contrast, we did not observe a Cbl-induced rise in [Ca^2+^]_i_ in CB chemoreceptor cells (Figure 5A), and Cbl did not cause a rise in catecholamine release, as would be predicted to occur if it was elevating [Ca^2+^]_i._ We also saw no effect of Cbl on catecholamine release to high K^+^ depolarization, whereas the response to high K^+^ in the rabbit CBs, where P/Q type channels are important in O_2_ sensing, is strongly attenuated by blockers of these channels [41]. This implies either that these channels do not contribute to voltage-dependent Ca^2+^ influx in the rat CBs (which is consistent with the observation that hypoxia-induced catecholamine release by rat CBs is reduced by ~95% with combined blockade of L- and T-type Ca^2+^ channels [42]) or that, if present, they are insensitive to Cbl. In addition, depletion of ER Ca^2+^ has no effect on catecholamine release in rat CBs, whereas this is virtually abolished by removal of extracellular Ca^2+^ [43], as is the hypoxia-induced rise in [Ca^2+^]_i_ (see Figure 6B) Thus, we do not believe that non-sulfide-related effects on CB Ca^2+^ handling were influencing the responses to Cbl which we observed.

The binding of exogenously applied sulfide by Cbl would be predicted to prevent it from having any effect on CB cells, and indeed this was confirmed by the results of Figure 1, which show that inclusion of 300 µM Cbl in the solution abolished the stimulation of ^3^H-CA release, which at the high concentration (200 µM) of NaHS we used in this experiment was probably due to the block of cytochrome C oxidase [14]. Similarly, application of Cbl abolished the rise in intracellular [Ca^2+^] induced in CB cells by 50 µM extracellular NaHS (Figure 5B).

The results in Figure 1C,D demonstrate that the slow-release sulfide donor GYY4137 also activated chemoreceptor cells, stimulating their release of newly synthesized catecholamines. This figure also shows that the presence of Cbl in the incubating solution fully eliminated the capacity of GYY4137 to activate chemoreceptor cells. Additionally, it can be observed in Figure 1C that application of Cbl by itself resulted in a significant inhibition of the basal ongoing release of catecholamines. This observation implies that Cbl might, upon its entry into chemoreceptor cells, be eliminating the H_2_S which is being produced under normoxic conditions, with the resulting inhibition of the baseline catecholamine-releasing activity of the cells. This interpretation, which would imply that endogenously generated sulfide is contributing to basal activity in chemoreceptor cells during normoxia, is consistent with the observation of Peng et al. [4] that knock out of the sulfide-synthesizing enzyme cystathionine-γ-lyase in mice caused a small but significant fall in ventilation even under normoxic conditions.

An important argument against a physiological role for sulfide, as assessed in many reports, is that the concentrations of its donors that are required to achieve effects are very high, so the presumable intracellular concentration of sulfide attained would exceed by far those resulting from endogenous production [13,14]. We therefore used GYY4137, which has not previously been employed in studies of the CBs, in an effort to generate a lower and sustained elevation of sulfide within the solution. Li et al. [36], in their original description of GYY4137, found that NaHS at a concentration of 100 µM in a phosphate buffer at pH 7.4 yielded amperometrically measured concentrations of H_2_S of 25,000 and 100,000 pA at 3 and 5 s, while GYY4137 at a concentration of 1 mM yielded sulfide concentrations of about 1800 and 3000 pA at 3 and 5 min. Using additional information from Li et al. [36], it can be estimated that adding 400 µM GYY4137 to 2 mL of solution would generate a concentration of 10–12 and ~25 µM of hydrogen sulfide at the end of a 10 and 20 min incubation, respectively. However, this calculation assumes no loss of sulfide from the solution, and although we attempted to slow the escape of H_2_S from our solutions by gassing the headspace in the vials (Figure 1B), the total sulfide concentration in the solution would probably have fallen rapidly and markedly during each 10 min incubation period because the solution was open to the atmosphere and gas was continually flowing through the headspace [44]. Moreover, sulfide is thought to enter cells as H_2_S, and based on the dependence of the equilibrium between H_2_S and HS^-^ on pH, temperature, and salinity of the Tyrode solution (see [45]), we calculate that pKa for this equilibrium would have been 6.45; in this case, the [H_2_S] would constitute only about ~10% of the total sulfide. These considerations would imply that the cellular H_2_S concentration in our experiments with GYY4137 was likely to have been in the submicromolar range, probably below the Ki of H_2_S to inhibit cytochrome oxidase [46]. Physiologically, the fact that the H_2_S degrading enzymes are mitochondrial while the H_2_S synthesis occurs largely in the cytoplasm [26] probably affords adequate protection to cytochrome oxidase against high rates of H_2_S production while allowing full range of cytoplasm and plasma membrane signaling by this gaseous messenger.

Whereas the effect of Cbl on basal ^3^H-CA release suggests that endogenous sulfide contributes to the setting of the functional activity of chemoreceptor cells under normoxic conditions, the data presented in Figure 2 and Figure 3 go a step further to indicate that endogenous sulfide plays a significant role as an amplifier of the chemoreceptor cell response to hypoxia. Importantly, Cbl significantly reduced ^3^H-CA release induced by three levels of hypoxia (2, 5, and 7% O_2_), in each case by >75%.

It is noteworthy that it took ~20 min for the effect of hypoxia on ^3^H-CA release to completely subside following the re-imposition of normoxia. The elevated release of catecholamine during this period was blocked if Cbl was present but was not significantly reduced if Cbl had been present during hypoxia but was removed when normoxia was restored (compare Figure 3A,D). These data suggest that a rise in cellular [sulfide] induced by hypoxia is likely to persist transiently after hypoxia has been removed, during which period it continues to promote catecholamine release. Since H_2_S diffuses rapidly across cell membranes due to its lipophilicity [44] such that cellular levels should drop very quickly if it is no longer being generated, we speculate that the persistence of the effect of sulfide could depend on a dynamic equilibrium between its binding to, and release from, cysteine residues on proteins [47]. By scavenging sulfide as it is released, Cbl would prevent its rebinding, thereby rapidly terminating signaling via its interactions with cysteine residues.

As shown in Figure 5, 50 µM sulfide caused an increase in [Ca^2+^]_i_ in glomus cells which was abolished by Cbl. In contrast, the rise in [Ca^2+^]_i_ evoked by high K^+^ depolarization was insensitive to Cbl. The effect of Cbl on the hypoxia-induced rise in [Ca^2+^]_i_ fell between these extremes; the Ca^2+^ signal was decreased by about 70%. The Ca^2+^ signal induced by both NaHS and hypoxia was abolished by removal of extracellular Ca^2+^, indicating that it was due entirely to Ca^2+^ influx (Figure 6A,B).

The L-type voltage-gated Ca^2+^ channel blocker nifedipine diminished the Ca^2+^ response to hypoxia by ~50%, compared to the 67% decrease reported previously [37]. The discrepancy between these values is relatively small and might be due to the use of different degrees of hypoxia, but in any case, it appears that a blockade of L-type voltage-gated Ca^2+^ channels causes a more substantial inhibition of the response to hypoxia than it does to that of sulfide, which was reduced by ~28%. CB chemoreceptor cells express several types of Ca^2+^ channels, although only the L-type channels seem to be required for the secretory response evoked by hypoxia in rat CBs because dihydropyridines (L-type channel inhibitors) virtually abolished the hypoxia-induced secretory response [42]. However, the T-type Ca^2+^ channel blocker mibefradil also strongly inhibited the secretory response to hypoxia, suggesting that Ca^2+^ entry through these channels may amplify the response to hypoxia. Interestingly, mibefradil had no effect on catecholamine release evoked by 35 mM K^+^ [42].

The partial sensitivity of the rise in [Ca^2+^]_i_ elicited by NaHS to nifedipine suggests that sulfide is stimulating the opening of both L- and T-type Ca^2+^ channels, with a predominant effect on the latter. T-type Ca^2+^ channels, which are not blocked by nifedipine, activate and inactivate at more negative potentials than do L-type channels. They therefore demonstrate a window current which develops with relatively small depolarizations, such as those associated with O_2_ sensing in the CBs [48,49] and may even be present at the resting potential [50,51]. Although we did not measure membrane potential, the K^+^ concentrations we used were substantial enough to ensure a robust depolarization, e.g., raising [K^+^]_e_ from 5 to 25 mM K^+^ would shift the K^+^ equilibrium potential by about +39 mV (assuming an intracellular [K^+^] of 130 mM). Thus, whereas T-channels could be active at the resting potential, such that their stimulation by basally generated sulfide causes low-level CB activation, they are likely to inactivate rapidly during high K^+^ depolarization, thereby accounting for the lack of effect of Cbl, and therefore sulfide, on catecholamine release by high K^+^.

The results of Figure 6 are in accord with the report by Makarenko et al. [8] that a significant component of the rise in [Ca^2+^]_i_ and consequent CB stimulation by hypoxia is due to a sulfide-mediated activation of T-type Ca^2+^ channels.

There is evidence that hypoxia-induced inhibition of both TASK and BK_Ca_ channels is important in CB O_2_ sensing [31,52]. Although sulfide does not appear to play a role in suppressing TASK channel activity during hypoxia [14,15,16], sulfide-induced BK_Ca_ channel inhibition has been observed in chemoreceptor cells, and the CBS antagonist AOAA inhibited the hypoxia-induced suppression of the BK_Ca_ current in rat type 1 glomus cells. This has led to the proposal that this mechanism is important in chemoreceptor O_2_ sensing [5,38,53]. An implication of this model is that a sulfide-mediated block of BK_Ca_ channels should contribute to the rise in chemoreceptor cell [Ca^2+^]_i_ triggered by hypoxia, but to date this seems not to have been examined. We therefore assessed whether the BK_Ca_ channel blocker IBTx would suppress the effect of 50 µM NaHS on [Ca^2+^]_i_ and found, as shown in Figure 6F, that it did not. Taken with the observation that Cbl strongly suppresses the hypoxia-induced [Ca^2+^]_i_ rise in these cells, these results do not support the concept that the involvement of sulfide in O_2_ sensing requires BK_Ca_ channel inhibition.

## 5. Conclusions

Our data demonstrate that Cbl is a useful tool for studying the function of endogenously produced sulfide, with which Cbl reacts and scavenges by forming the stable compound sulfhydrylcobalamin. It penetrates into cells and upon its reaction irreversibly eliminates the gaseous messenger, regardless of how it is being produced. Thus, while it is uncertain that inhibitors or knockout of individual sulfide-producing enzymes will completely eliminate increases in cellular [sulfide] during hypoxia, and there are also worries about the marked rise in plasma homocysteine which occurs in CSE knockout mice [27], Cbl would be expected to abolish a rise in sulfide, whatever its cause, without causing cell toxicity. Our use of this approach has allowed us to provide compelling and novel evidence supporting the hypothesis that endogenous sulfide contributes to O_2_ sensing in CB chemoreceptor cells and is capable of regulating their activity, both under basal conditions and during natural hypoxic stimulation. Our evidence supports the possibility that this contribution is due to the opening of T-type Ca^2+^ channels, as demonstrated previously by Makarenko and colleagues [8]. Thus, endogenously generated sulfide would act as a positive messenger, enhancing the gain of the hypoxic transduction cascade, especially at low to moderate levels of hypoxia.
